# ILLNESS AND THE LANGUAGE OF POETRY

**DOI:** 10.1093/geroni/igz038.2105

**Published:** 2019-11-08

**Authors:** Destiny Birdsong

**Affiliations:** Vanderbilt University, Nashville, Tennessee, United States

## Abstract

Two interactive workshops were conducted: one for training healthcare professionals in nursing, PT, and pharmacy, and another for the continuing education for caregivers of HIV-positive patients. Active Listening and Visual Learning Strategies for Healthcare Professionals discussed the importance of using active listening skills to better hear, understand, and treat patients. Activities included viewing one WOE respondent’s artwork and hearing a clip of the respondent’s interview, followed by a facilitated discussion of how the respondent’s story about her childhood shed light on her art. Additionally two published poems about bodies and illness were shared, with discussion of how each poem’s circuitous narrative nature offered clues about the speaker’s illness and outlook on life. Cultural Humility’s activities focused on utilizing poetry to discuss how caregivers can use active listening strategies to better understand how patients cope with life-changing illnesses, and how they incorporate the challenges of those illnesses into rich, fulfilling lives.

